# Determining the impact of vaccination on SARS-CoV-2 RT-PCR cycle threshold values and infectious viral titres

**DOI:** 10.1099/acmi.0.000597.v3

**Published:** 2023-10-20

**Authors:** Katherine L. Peterson, Julia P. Snyder, Hannah W. Despres, Madaline M. Schmidt, Korin M. Eckstrom, Allison L. Unger, Marya P. Carmolli, Joseph L. Sevigny, David J. Shirley, Julie A. Dragon, W. Kelley Thomas, Emily A. Bruce, Jessica W. Crothers

**Affiliations:** ^1^​ Department of Medicine, University of Vermont Medical Center, Burlington, VT, 05405, USA; ^2^​ Department of Pathology and Laboratory Medicine, Robert Larner, M.D. College of Medicine, University of Vermont, Burlington, VT, 05405, USA; ^3^​ Department of Microbiology and Molecular Genetics, Robert Larner, M.D. College of Medicine, University of Vermont, Burlington, VT, 05405, USA; ^4^​ Hubbard Center for Genome Studies, University of New Hampshire, Durham, NH, 03824, USA; ^5^​ Faraday, Inc. Data Science Department, Burlington, VT, 05405, USA

**Keywords:** C_T_, COVID-19, micro-focus forming assay, RT-qPCR, SARS-CoV-2, viral titre

## Abstract

**Background.:**

As the COVID-19 pandemic continues, efforts to better understand severe acute respiratory syndrome coronavirus 2 (SARS-CoV-2) viral shedding and transmission in both unvaccinated and vaccinated populations remain critical to informing public health policies and vaccine development. The utility of using real time RT-PCR cycle threshold values (C_T_ values) as a proxy for infectious viral litres from individuals infected with SARS-CoV-2 is yet to be fully understood. This retrospective observational cohort study compares quantitative infectious viral litres derived from a focus-forming viral titre assay with SARS-CoV-2 RT-PCR C_T_ values in both unvaccinated and vaccinated individuals infected with the Delta strain.

**Methods.:**

Nasopharyngeal swabs positive for SARS-CoV-2 by RT-PCR with a C_T_ value <27 collected from 26 June to 17 October 2021 at the University of Vermont Medical Center Clinical Laboratory for which vaccination records were available were included. Partially vaccinated and individuals <18 years of age were excluded. Infectious viral litres were determined using a micro-focus forming assay under BSL-3 containment.

**Results.:**

In total, 119 specimens from 22 unvaccinated and 97 vaccinated individuals met all inclusion criteria and had sufficient residual volume to undergo viral titring. A negative correlation between RT-PCR C_T_ values and viral litres was observed in both unvaccinated and vaccinated groups. No difference in mean C_T_ value or viral titre was detected between vaccinated and unvaccinated groups. Viral litres did not change as a function of time since vaccination.

**Conclusions.:**

Our results add to the growing body of knowledge regarding the correlation of SARS-CoV-2 RNA levels and levels of infectious virus. At similar C_T_ values, vaccination does not appear to impact an individual’s potential infectivity when infected with the Delta variant.

## Data Summary

Patient-level metadata includes identifiers and is available upon request following ethical review. The sequencing data were deposited to the NCBI Sequence Read Archive under the BioProject PRJNA938406 and accession numbers are found in Table S1, available in the online version of this article.

## Introduction

The COVID-19 pandemic continues to cause significant morbidity and mortality worldwide. As of January 2023, 6.7 million deaths have been attributed to severe acute respiratory syndrome coronavirus 2 (SARS-CoV-2) [1]. The arrival of effective vaccines, including both viral vectored and mRNA COVID-19 vaccines, in late 2020, led to a significant decline in symptomatic disease, hospitalizations and mortality among vaccinated individuals [2–4]. Initially, vaccine breakthrough infections were uncommon [5]. However, the emergence of the highly transmissible Delta variant considerably altered the trajectory of the pandemic. While vaccines continued to offer robust protection against hospitalization and death, vaccine breakthrough infections became increasingly common [2, 3, 6, 7]. Efforts to better understand SARS-CoV-2 viral shedding and transmission in both unvaccinated and vaccinated populations are critical to inform public health policies and vaccine development but continue to be complicated by the emergence of new variants.

Often, real time RT-PCR cycle threshold (C_T_) values are used as a proxy for infectious viral litres in both clinical and research settings and multiple studies have demonstrated that lower C_T_ values (representing higher viral RNA levels) positively correlate with an increased probability of isolating SARS-CoV-2 in viral culture [8–11]. This is supported by epidemiologic studies that report an association between lower C_T_ values and an increased risk of onward household transmission [12–15]. However, while culture-based studies are typically unable to isolate live virus beyond 8–10 days following the onset of symptoms, detection of viral RNA by RT-PCR can extend for weeks, even months, post-symptomatology [9–11, 16, 17].

Several key limitations exist when utilizing C_T_ values as a proxy for infectivity. First, although detection of SARS-CoV-2 RNA by RT-PCR is the gold standard for the diagnosis of COVID-19 disease, it is unable to differentiate between infectious and non-infectious viral particles, thereby potentially overestimating infectivity [11]. Second, many clinical and pre-analytic variables may impact RT-PCR results, including time since symptom onset, specimen collection method and source, and processing timeline [18]. Third, there are neither Food and Drug Administration-approved quantitative RT-PCR assays nor universal standards widely available to establish comparable calibration curves, making it difficult to interpret C_T_ results across different assay platforms, laboratories and studies [18]. Lastly, viral culture traditionally provides qualitative rather than quantitative results, providing information regarding the presence or absence of infectious virus but not allowing for comparison of precise levels of virus between clinical samples. In addition, viral culture is currently not widely performed in clinical or research laboratories due to biosafety and technical challenges.

Despite the critical public health importance, relatively little is understood about how vaccination against SARS-CoV-2 impacts viral infectivity during acute infection. Data during Alpha variant circulation showed that RT-PCR C_T_ values were higher in vaccinated individuals compared to those who were unvaccinated and that vaccination reduced onward transmission of the Alpha variant in household contacts [6, 7, 19–22]. Eyre *et al*. showed similar findings, however to a lesser extent, when infection was with the Delta variant compared to the Alpha variant [12]. Whether the relationship between C_T_ values and infectivity is different in vaccinated versus unvaccinated individuals is also poorly understood. If true, this knowledge would significantly impact the interpretation of C_T_ values in the clinical setting.

Here we present data from a retrospective observational cohort study utilizing a high-throughput focus-forming viral titre assay to compare quantitative infectious viral litres across a range of SARS-CoV-2 RT-PCR C_T_ values in both unvaccinated and vaccinated individuals infected with the Delta strain of the SARS-CoV-2 virus. While continued evolution of the SARS-CoV-2 virus has resulted in circulation of additional strains since Delta, these results provide critical information regarding the correlation of RNA levels and infectious virus and offers insight into the impact of vaccination on SARS-CoV-2 infectivity as new variants continue to emerge.

## Methods

### Study design and setting

The University of Vermont Medical Center (UVMMC) is an academic medical centre located in Burlington, Vermont. It is the only tertiary referral centre in the state. The hospital laboratory processes both inpatient and outpatient specimens. Nasopharyngeal (NP) swabs positive for SARS-CoV-2 by RT-PCR with a C_T_ value <27 collected from 26 June to 17 October 2021 for which vaccination records were available were included. This cut off was chosen because we demonstrated in prior work that infectious virus was unable to be isolated by this method from specimens with C_T_ values >27^23^. Partially vaccinated individuals and individuals <18 years of age were excluded. We considered an individual to be fully vaccinated against COVID-19 if 14 days or more had passed since completion of a primary vaccination series of either two-doses of an mRNA vaccine (Pfizer or Moderna) or a single-dose of a viral vector vaccine (J and J’s Janssen). Vaccination data was derived from both the Vermont Department of Health Vaccine Registry and the UVMMC Electronic Medical Record. Inclusion dates were selected based on publicly available epidemiologic data of the prevalence of circulating SARS-CoV-2 variants in the region. During this time, the Delta variant comprised the overwhelming majority of circulating cases (>90 % according to outbreak.info data of SARS-CoV-2 cases sequenced in Vermont).

### Ethical approval

The University of Vermont’s Institutional Ethical Review Board approved this study under a waiver of consent (CHRMS STUDY00000881).

### Specimen collection and storage

From March 2020 through October 2021, all respiratory specimens with sufficient residual volume that tested positive for SARS-CoV-2 by RT-PCR at the UVMMC Clinical Laboratory were coded and transferred from refrigeration to −80°C for long-term storage within 4 days of initial testing. In accordance with clinical laboratory specimen requirements and procedures, all samples remained refrigerated from the time of specimen collection until being transferred to long-term storage. Samples used in this study represent a subset of this larger sample set.

### Viral RNA quantification by RT-PCR

**Fig. 1.**
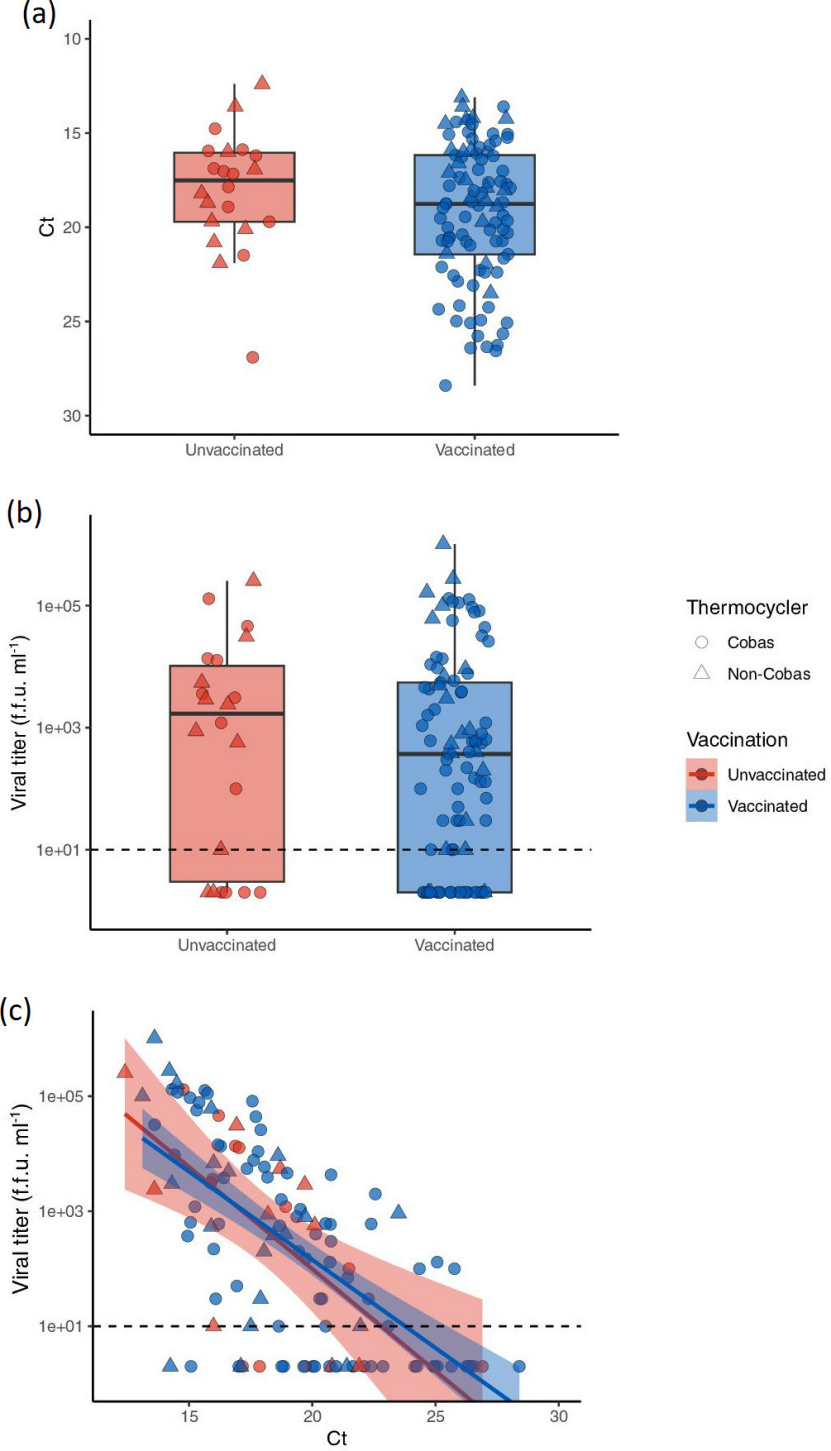
Infectious viral titres and C_T_ values for unvaccinated versus vaccinated individuals infected with Delta variant. (a) Viral RNA (C_T_) versus vaccination status. Data are summarized by boxplots and overlaid with points representing individual subjects. The *y* axis is flipped for visualization as C_T_ values are inversely proportional to the amount of viral RNA. (b) Viral titre (f.f.u. ml^–1^) by vaccination status. Data are summarized by boxplots and overlaid with points representing individual subjects. Dashed line indicates the limit of detection for infectious titer (10 f.f.u. ml^–1^). (c) Viral RNA (C_T_) on the *x* axis plotted against viral titre (f.f.u. ml^–1^) on the *y* axis. Separate linear regression lines (*ŷ* = *β*0 + *β*1*x̄* + *Ɛ*) were fit to unvaccinated and vaccinated individuals. Shading indicates confidence interval (0.95) for each line. (a−c) Red symbols and lines indicate unvaccinated individuals (*N* = 12), blue symbols and lines indicate vaccinated individuals (*N* = 76). Thermocycler method is indicated by shape (Cobas circles, non-Cobas triangles). f.f.u. stands for focus forming unit.

**Fig. 2.**
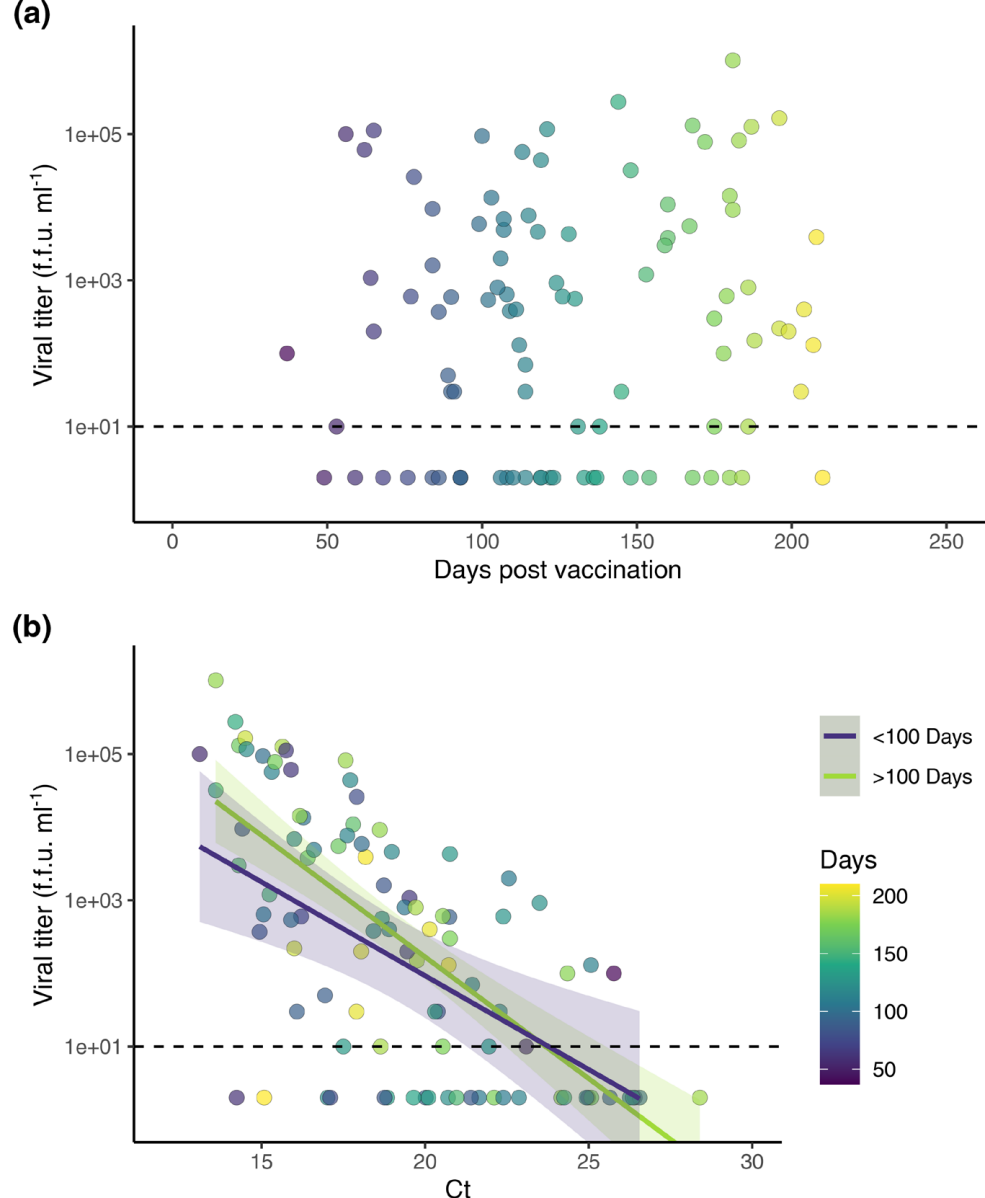
Infectious viral titres and C_T_ values as a function of time since vaccination. Clinical specimens from vaccinated individuals (*N* = 97) infected with SARS-CoV-2 Delta variant were used to visualize the relationship between viral titer, viral RNA (C_T_), and time since full vaccination. (a) Days since fully vaccinated (≥14 days since completion of a primary COVID-19 vaccine series) on the *x* axis plotted against viral titer (f.f.u. ml^–1^) on the *y* axis. (b) Viral RNA (C_T_) on the *x* axis plotted against viral titre (f.f.u. ml^–1^) on the *y* axis. Data point fill color corresponds with days post vaccination. Samples were grouped by <100 days (purple) or >100 days (green) post vaccination, and separate linear regression lines (*ŷ* = *β*0 + *β*1*x̄* + *Ɛ*) were fit to each group. Shading indicates confidence interval (0.95) for each line. (a, b) Dashed line indicates the limit of detection for infectious titer (10 f.f.u. ml^–1^). f.f.u. stands for focus forming unit.

The UVMMC Clinical Laboratory routinely utilized four RT-PCR assays for diagnostic testing during the study period: ABI Quantstudio Flex 7 (Thermo Fisher Scientific), Cobas 6800 (Roche), GeneXpert (Cepheid) and Panther Fusion (Hologic). Gene targets and analytic testing characteristics vary across the different platforms with the ABI Quantstudio assay targeting the N1 and N2 genes, the Cobas targeting the E and ORF1ab genes, GeneXpert targeting the E and N2 genes, and the Panther Fusion targeting two conserved regions of the ORF1ab sections of the SARS-CoV2 genome. All assays had received Emergency Use Authorization (EUA) approvals and were performed in a diagnostic clinical laboratory in accordance with Clinical Laboratory Improvement Amendments (CLIA) standards. In order to minimize variability in reported C_T_ values, we reran samples with sufficient volume originally tested on the ABI Quantstudio, GeneXpert, or Panther Fusion on the Cobas 6800. Fig. 1 indicates whether reported C_T_ values are derived from ‘Cobas’ or ‘Non-Cobas’ platforms, but Fig. 2 does not make this distinction.

### Focus-forming assay

All SARS-CoV-2 viral titring was conducted at the University of Vermont BSL-3 facility, under an approved IBC protocol, as previously published by Despres *et al*. [23]. Clinical samples were titred using a microfocus-forming assay on VeroE6-TMPRSS2 cells (Japanese Cancer Research Resources Bank No. JCRB1819). Cells were seeded in a white bottom 96-well plate (Falcon, Cat. #353296), 24 h before infection (60 000 cells/well). Samples were serially diluted in DMEM (Gibco, Cat. No. 11965084) using tenfold dilutions. All samples were titred in duplicate across two serial dilutions, with undiluted sample titred in a single well due to limitations of specimen volume. Cells were infected for 1 h at 37 ℃, overlayed with 1.2 % methylcellulose (Acros, Cat. No. 332620010) in DMEM and incubated for 24 h at 37℃. Cells were fixed using 4 % formaldehyde in PBS, permeabilized using 0.01 % Triton X-100 in PBS (15 min) and blocked (5 % dry milk in PBS) for 1 h before incubated in a primary, cross-reactive rabbit anti-SARS-CoV N monoclonal antibody (Sino Biological, Cat. No. 40143R001) at 1 : 20 000 dilution for an additional hour. Wells were washed in PBS, incubated with a peroxidase-labelled goat-anti-rabbit antibody (Seracare, Cat. No. 5220–0337) at 1 : 4000 for 1 h and developed using a peroxidase substrate (SeraCare, Cat. No. 5510–0030).

### Whole-genome sequencing

A limited number of specimens were randomly selected for whole-genome sequencing (WGS), which was performed by the Hubbard Center for Genome Studies at the University of New Hampshire. Briefly, coded samples were shipped to UNH on dry ice where the COVID-19 ARCTIC v3 primer panel and the Illumina COVIDSeq RUO kit protocol (1000000126053 v06) was used to construct Illumina sequencing libraries. Whole-genome sequencing was performed on the NovaSeq 6000 Sequencing System (Illumina, San Diego, USA) and produced 250 bp paired-end reads. Sample datasets were demultiplexed, filtered for known sequencing contaminants, and consensus genome sequences were constructed using a reference-based mapping approach (Wuhan-Hu-1 reference sequence NC_045512.2) within the BaseSpace Labs DRAGEN COVID Lineage application v3.5.2. The software performs Kmer-based SARS-CoV-2 detection and then aligns the sequencing reads against the reference genome to perform variant calling and consensus sequence generation. The sequencing data were deposited to the NCBI Sequence Read Archive under the BioProject PRJNA938406 and accession numbers are found in Table S1.

### Data sources

Patient-level data including test order details, patient demographics, vaccination history, and medical history, were extracted from the UVMMC Electronic Medical Record. Additional COVID-19 vaccination data were obtained with permission from the Vermont Department of Health Immunization Registry.

### Statistical analysis

Comparisons between groups were performed using two-tailed unpaired *t*-test for continuous and Chi-square for categorical variables. Statistical tests used for comparisons of RT-PCR Ct values included Welch two sample *t*-test (two-tailed) and exact two sample Kolmogorov–Smirnov (two-sided) for viral titrer. Linear regression models (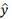
= β0 + β1 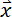 + Ɛ) were fit to the viral litres and C_T_ values data and shading represents the confidence interval (0.95). Statistical analyses were performed using GraphPad Prism (9.5.0) and R Studio (4.2.1).

## Results

### Descriptive characteristics

**Fig. 3.**
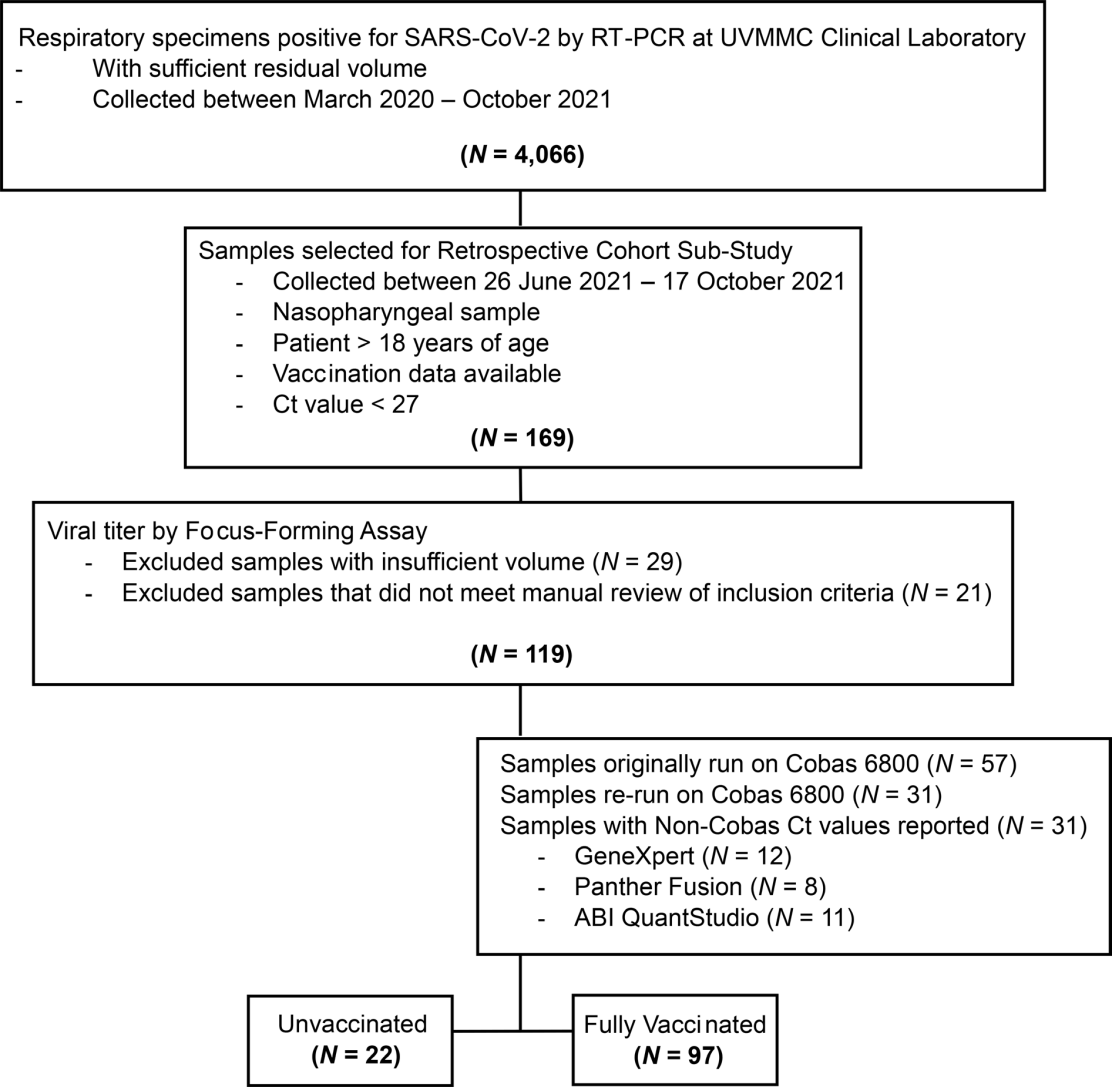
CONSORT diagram. Flow chart of clinical specimens included for analysis.

From March 2020 to October 2021, 4066 clinical respiratory specimens positive for SARS-CoV-2 by RT-PCR were captured. Of these, 169 available specimens were identified that met all inclusion and exclusion criteria and were collected during the time period of interest (26 June to 17 October 2021). Viral litres were successfully performed in 119 samples; 29 specimens were unable to be titred due to insufficient volume. C_T_ values were initially derived from RT-PCR platforms other than the Cobas 6800 for 62(52.1 %) specimens. Following viral titring, sufficient volume was available to allow for repeat testing on the Cobas 6800 in 31 of these samples (Fig. 3). Ultimately, data derived from 119 specimens remained for analysis, of which 22(18.5 %) were from unvaccinated individuals and 97(81.5 %) were from fully vaccinated individuals.

The demographic characteristics of the individuals included in our analysis are shown in Table 1. The mean age and male:female ratio were similar between groups. In the vaccinated group, the mean number of days from the time individuals were considered fully vaccinated (14 days following the last dose of their primary series) to the collect date of their positive test was 128.3 with a range of 37 to 210 days. Booster shots were not authorized until towards the end of the study period and none of the individuals included in our analysis had yet been boosted at the time of their positive test. Of the 97 specimens from vaccinated individuals, 86(88.7 %) received an mRNA vaccine versus 11(11.3 %) who received Janssen/Johnson and Johnson, a viral vector vaccine. Within the mRNA vaccine group, 65(75.6 %) individuals were vaccinated with Pfizer/BioNTech versus 21(24.4 %) with Moderna.

**Table 1.** Cohort characteristics. The statistical tests for age in year (unpaired *t*-test, two-tailed) and sex (Chi-square, two-sided) were performed in GraphPad (9.5.0). The statistical tests for RT-PCR Ct value (Welch two sample *t*-test, two-tailed) and viral titre (exact two sample Kolmogorov–Smirnov, two-sided) were performed in R Studio (4.2.1)

	Unvaccinated SARS-CoV-2 cases	Vaccinated SARS-CoV-2 cases	*P* value
Number of specimens, *n* (%)	22 (18.5)	97 (81.5)	–
Age in years, mean (range)	46.1 (20–73)	48.2 (18–95)	0.6408
Female sex, *n* (%)	11 (50)	55 (56.7)	0.5680
Male sex, *n* (%)	11 (50)	42 (43.3)	
RT-PCR Ct value, mean (range)	18 (12.4–26.9)	19.2 (13.1–28.4)	0.1321
Viral titre (f.f.u. ml^–1^), mean (range)	23116.4 (0–255000)	27266.3 (0–1020000)	0.2602
Days since full vaccination, mean (range)	–	128.3 (37–210)	–
Vaccine received			
Viral vector vaccine, *n* (%)	–	11 (11.3)	–
mRNA vaccine, *n* (%)	–	86 (88.7)	–
mRNA manufacturer			
Moderna, *n* (%)	–	21 (24.4)	–
Pfizer/BionTech, *n* (%)	–	65 (75.6)	–

f.f.u, focus forming unit.

All SARS-CoV-2 positive specimens included in this study were presumed to be the Delta variant based on publicly available genomic data, which demonstrated that Delta was the predominate variant in circulation in Vermont during the study period as noted above. This was corroborated by WGS performed on a subset of 37 randomly selected samples; all of which were identified as the Delta variant.

### C_T_ values and viral litres are similar in unvaccinated and vaccinated individuals infected with the Delta variant

The mean C_T_ value observed in vaccinated individuals was 18.0 with a range of 12.4 to 26.9 while the mean value in unvaccinated individuals was 19.2 with a range of 13.1 to 28.4. The mean direct viral titre obtained from NP specimens collected from fully vaccinated individuals was 23 116.4 f.f.u. ml^–1^ (range: 0.0 to 255 000 f.f.u. ml^–1^) while the mean from unvaccinated individuals was slightly increased at 27 266.30 f.f.u. ml^–1^ (range: 0.0 to 1 020 000 f.f.u. ml^–1^). No significant differences were observed in mean C_T_ values (*P*=0.1321) or direct viral litres (*P*=0.2602) between groups (Table 1, Fig. 2a, b). To assess for the potential impact of outliers, we also compared the overall proportion of unvaccinated versus vaccinated individuals with undetectable litres and found no difference in the overall presence of titrable virus between the groups (undetectable viral litres were observed in 27.3 % of unvaccinated vs. 27.8 % of vaccinated samples; Table 2).

**Table 2.** Proportion of samples from unvaccinated versus vaccinated individuals with viral litres below and above the limit of detection. Detectable viral litres (f.f.u. ml^–1^) are greater than or equal to the assay limit of detection (10 f.f.u. ml^–1^)

	Unvaccinated	Vaccinated
Undetectable titre, *n* (%)	6 (27.3)	27 (27.8)
Detectable titre, *n* (%)	16 (72.7)	70 (72.2)
Total	22 (100)	97 (100)

f.f.u, focus forming unit.

### RT-PCR CT values broadly correlate with viral litres in both unvaccinated and vaccinated individuals

We generally found that direct viral litres increased as C_T_ values decreased (signifying higher levels of viral RNA), resulting in a negative correlation between RT-PCR CT values and direct viral infectious litres. This correlation held in both unvaccinated and vaccinated individuals infected with the Delta variant as demonstrated in Fig. 2c. Additionally, the likelihood of being able to titre virus from a sample decreased as the C_T_ value increased. We were unable to titre virus from any samples with a C_T_ >26. Conversely, infectious virus was titrable from the majority [86.7 %(65/75)] of samples with C_T_ values <20. However, in agreement with prior studies, we observed variation at the patient level between the amount of RNA and the amount of infectious virus present, suggesting that C_T_ is an imprecise measurement of infectious viral load [8, 23, 24].

### Viral litres do not change as a function of time since vaccination

To evaluate whether there exists a time-dependent effect of vaccination on infectivity, we assessed direct viral litres as a function of time since vaccination (Fig. 3a). No correlation was observed. We also compared the relationship between viral litres and C_T_ values as a function of time since vaccination using a linear regression model and observed no differences (Fig. 3b).

Lastly, separate linear regression lines were fit to cases occurring within 100 days versus >100 days post-vaccination and revealed no difference in the relationship between infectious viral litres and C_T_ values between these two groups.

## Discussion

In this study we assessed the impact of vaccination status on viral RNA levels (as measured by RT-PCR) and quantitative viral litres in individuals infected with the Delta variant of SARS-CoV-2. We found a negative correlation between C_T_ values and direct viral litres obtained using a focus-forming assay in individuals infected with the Delta variant and were unable to titre virus from any samples with a C_T_ >26, findings which are in keeping with our earlier work [23]. While this suggests that C_T_ values may serve as a reasonable proxy for infectiousness at a population level, our data also revealed significant individual variation in viral titre levels across C_T_ values and undetectable viral litres in 13 %(10/75) of individuals with a C_T_ <20. The reason for this is unclear and could be related to a combination of both biologic (differences in viral inoculum, time since symptom onset, immune status of host, etc.) and preanalytical variables (time from sample collection to accessioning, sample storage, etc.). Ke *et al*. also demonstrated significant variability of viral dynamics among individuals, and therefore, caution should still be used when making clinical decisions based on C_T_ values alone [25]. While there is a large body of evidence indicating infectious virus is unlikely to be cultured from samples with a C_T_ >30, caution should also be used when inferring infectivity of patients based on individual C_T_ values, particularly when complicating factors such as immune-suppression or antiviral drugs (i.e. nirmatrelvir-ritonavir) are present.

Importantly, we observed no difference in mean C_T_ value or direct viral titre between vaccinated and unvaccinated groups and vaccination status did not impact the relationship between C_T_ value and viral litres of individuals infected with the Delta variant. Additionally, no difference in the proportion of individuals with undetectable viral litres was observed between vaccinated and unvaccinated individuals, suggesting that vaccination status alone does not impact the likelihood of viral recovery from a given sample. Few studies have quantified SARS-CoV-2 infectious viral litres using a focus-forming assay, and this study, to our knowledge, is only the second of its kind to compare direct viral litres in unvaccinated and vaccinated individuals [9, 24]. Similar to our study, Puhach *et al*. evaluated infectious viral litres of NP swabs with RT-PCR C_T_ values <27 from unvaccinated and vaccinated individuals infected with the Delta variant [24]. In agreement with our results, they found that while more RNA broadly equated to more infectious virus in both unvaccinated and vaccinated individuals, the correlation of this trend was weak, with significant variation in the ratio of RNA to virus between individuals [24]. They also reported a 2.8-fold decrease in viral genome copies calculated from C_T_ value RNA levels following vaccination; however, the clinical and epidemiologic relevance of the reported difference is unclear [24]. While we had anticipated a potential differential impact of vaccination status on litres versus C_T_ values due to the neutralizing capabilities of vaccine-induced antibodies, our findings suggest that receipt of original SARS-CoV-2 vaccine formulations does not markedly decrease the potential infectivity of an individual infected with the Delta variant. This is in contrast to earlier data that reported reductions in both C_T_ values and onward transmission of pre-Delta strains following vaccination [12, 13, 15, 19, 21, 22, 26, 27]. With widespread circulation of the Delta variant in the summer of 2021, reports of similar C_T_ values in vaccinated and unvaccinated cohorts began to emerge, raising concern regarding the impact of vaccination on infectivity and transmission [6, 7, 12, 28]. Reports of lower infectious viral litres at similar C_T_ values and reductions in the duration of viral shedding in vaccinated individuals provided some hope that vaccination was still capable of limiting widespread community spread of the virus; however, additional epidemiologic studies ultimately revealed similar secondary attacks rates in vaccinated and unvaccinated populations infected with the Delta variant [29–32]. We believe that these data provide additional *in vitro* evidence that the emergence of Delta and subsequent SARS-CoV-2 variants have ameliorated the impact of vaccination on viral infectivity and subsequent community transmission and supports the continued use of additional public health interventions to mitigate viral transmission. Importantly, these data indicate a clear need for the development of second-generation vaccines targeting mucosal immune responses to reduce viral transmission.

Further, we did not observe any changes in C_T_ values or viral litres as a function of time since vaccination suggesting that there is no waning effect of vaccination on Delta transmission potential. This is in contrast to several other studies that have demonstrated waning of vaccine-associated reductions in RNA viral load at 6 months post-vaccination [27]. It is possible that our study does not include the necessary post-vaccination time range to observe such changes (mean days since vaccination, 128.3 days) and that our sample size is too small to detect meaningful differences in the relationship between C_T_ values and viral litres across our study population.

Our study has at least three notable limitations. First, we lacked some sample-associated metadata, including time since symptom onset. Although we were able to extract data from our institution’s electronic medical record, most individuals were tested in the outpatient setting, and the test order did not include any details regarding the presence, absence or duration of symptoms. Therefore, we were unable to correlate our data with days post-onset of symptoms or control for differences in symptom status in vaccinated versus unvaccinated patients. This may bias our results and should be considered when interpreting these data. Singanayagam *et al*. demonstrated that vaccinated individuals reach similar peak viral loads as unvaccinated individuals but have a more rapid decline in viral load, which could have a significant impact on onward transmission dynamics at the population level [29]. Considering when in the disease course testing is performed is therefore of critical interest. Additionally, it is possible that differences in test-seeking behaviour existed between groups such that unvaccinated persons sought diagnostic testing sooner in their disease course thus skewing the data towards higher viral loads in this group. Second, due to limited residual specimen volume, we were unable to obtain C_T_ values using the Cobas platform on all specimens, which may have impacted the comparability of C_T_ values across our data set. Third, our results are limited by a small sample size and an uneven distribution of specimens from unvaccinated versus vaccinated individuals, with only 18.5 % of cases occurring in unvaccinated individuals. While this was not surprising considering that early vaccination efforts resulted in Vermont being the first state in the United States to reach national vaccination targets and vaccination rates continue to be amongst the highest in the country, it limited the number of unvaccinated cases available for comparison and thus constrained robust statistical comparisons between the groups.

Despite these limitations, this study contributes to a growing body of literature examining the relationship between C_T_ values and viral infectivity as well as the impact of vaccination on SARS-CoV-2 transmission potential. Since the time of this work the Delta variant has been surpassed by other strains, most notably those of the Omicron lineages, and viral evolution continues to impact the effect of currently available vaccines. Caution however should continue to be used when using clinical testing data (e.g. C_T_ values) to infer infectivity of individual patients, regardless of vaccination status and viral strain. Continued generation of quantitative viral culture data is critical to enhance our understanding of the impact of novel viral strains and vaccine formulations on viral transmission potential and to inform effective public health interventions.

## Supplementary Data

Supplementary material 1
