# Supplementary material for: Determining the impact of vaccination on SARS-CoV-2 RT-PCR cycle threshold values and infectious viral titres
Source: Access Microbiol. 2023 Oct 20;5(10):000597.v3. doi: 10.1099/acmi.0.000597.v3 (PMC10634488; doi:10.1099/acmi.0.000597.v3)
Supplement: Supplementary material 1 [file acmi-5-597.v3-s001.pdf]

Supplementary Table 1. Sequencing data were deposited to the NCBI Sequence Read Archive under the BioProject PRJNA938406 with the following accession numbers.

Collection Date	Deidentified ID	SRA Accession	BioSample Accession
2021-08-26	969e16c9-541a-42b3-a217-651f0b524a73	SRR23612258	SAMN33434917
2021-08-25	399030d6-1020-4678-a086-2220a131f526	SRR23612267	SAMN33434909
2021-08-26	de2aefa1-054e-473b-ba6c-22d0a75bc4e6	SRR23612260	SAMN33434915
2021-08-26	6ad4b475-ccb3-4139-9796-fafd5031d321	SRR23612274	SAMN33434903
2021-08-26	28bb72f2-8190-425d-8a3d-cf0ce5955f2d	SRR23612275	SAMN33434902
2021-08-28	7f1713bd-1f14-4320-b14c-7d3ebff430a9	SRR23612277	SAMN33434900
2021-09-03	74097e26-3ce2-41ca-a4bf-67019265576f	SRR23612257	SAMN33434918
2021-09-04	9800ec12-2d2c-40d2-916d-ccca7909a917	SRR23612266	SAMN33434910
2021-09-05	a8e44555-78ff-44b1-a7f4-5c1e6385c6dc	SRR23612256	SAMN33434919
2021-09-04	a3896a3c-c013-4e30-8e3d-4d35010e5cd1	SRR23612263	SAMN33434913
2021-09-01	28b7d05b-4d75-430e-a2f2-e643a972c3f5	SRR23612259	SAMN33434916
2021-09-01	36bb4e9b-9776-4ce7-add7-3a50449c05f2	SRR23612261	SAMN33434914
2021-09-01	6526622d-46ba-49c7-b372-2af5c435e6da	SRR23612265	SAMN33434911
2021-09-01	c058757c-a0cf-423f-b103-df17959452cc	SRR23612255	SAMN33434920
2021-08-05	3a01f632-57d9-479d-877b-35fae982eecb	SRR23612264	SAMN33434912
2021-08-07	ec6f266d-6eef-471b-8049-57e49e4a3aa1	SRR23612269	SAMN33434907
2021-07-23	80a49d9a-eb64-46ea-98f2-33c025f979b0	SRR23612272	SAMN33434904
2021-07-24	ea07e6a3-6f16-4e40-bb69-03ab9bf827f1	SRR23612268	SAMN33434908
2021-07-24	c4832004-23c9-4e91-8320-4115452476a9	SRR23612271	SAMN33434905
2021-07-27	f305c926-2cdb-4787-b2cf-599e11ae01a4	SRR23612276	SAMN33434901
2021-07-28	e1127d69-2b62-4d7f-90e3-c4275ff594e9	SRR23612270	SAMN33434906
2021-07-31	e9a50cd4-f64f-41db-8cee-24f09f7cde5f	SRR23612279	SAMN33434898
2021-07-19	3ed7530f-8036-4ac3-b102-fc4166c7947a	SRR23612282	SAMN33434895
2021-08-24	25cb41f4-c711-48cd-b923-8d0cd188b2d4	SRR23612281	SAMN33434896
2021-07-29	b783ac1d-ba20-401d-a6a4-f2c571e34679	SRR23612249	SAMN33434893
2021-07-29	0cdf5423-39b3-46e3-b447-c8465c17b734	SRR23612251	SAMN33434891
2021-07-30	cc0c5214-30bf-4bb6-8912-e8bc623d055a	SRR23612280	SAMN33434897
2021-07-29	6350efbf-9d13-4320-8b07-82192de743e5	SRR23612278	SAMN33434899
2021-08-02	bf05c0a7-dec7-4d20-a2a1-018bd72baa00	SRR23612250	SAMN33434892
2021-08-03	ee588edf-9f7c-45ff-8701-03038c5e9300	SRR23612283	SAMN33434894
2021-08-02	4c571822-c062-4e60-83eb-b36e7dab509a	SRR23612262	SAMN33434887
2021-08-02	b3c1e65b-b06d-4717-b5e8-12d836193342	SRR23612252	SAMN33434890
2021-08-04	8c7d8414-84df-4489-80b7-584b8aafa6bd	SRR23612254	SAMN33434888
2021-08-04	786808c8-92cc-470b-9211-e6ee57afba1c	SRR23612285	SAMN33434884
2021-08-03	42e1b35b-5bab-43b0-a765-eb412a36a42c	SRR23612253	SAMN33434889
2021-08-04	ea784cf3-21c3-4a91-ba1f-4a641184927e	SRR23612284	SAMN33434885
2021-08-03	a537fc2d-b6ed-455c-85cb-a16a50d0302c	SRR23612273	SAMN33434886
